# David Gregor (Greg) Wilkinson, MD, FRCP (Ed), FRCPsych

**DOI:** 10.1192/bjb.2023.31

**Published:** 2023-10

**Authors:** Christine Wilkinson

Formerly Professor of Liaison Psychiatry, University of Liverpool Medical School, UK

**Figure:**
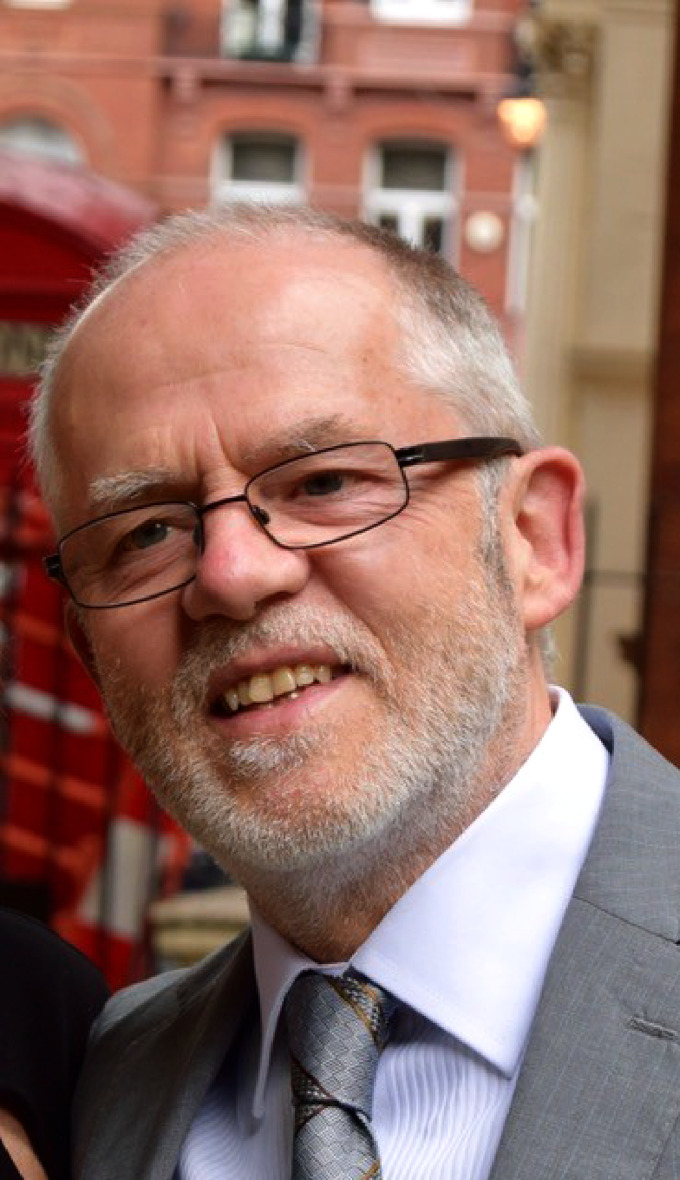

Greg Wilkinson, who died on 15 January 2023 at the age of 71, was Editor of the *British Journal of Psychiatry* from 1993 to 2003. As Editor, his stated aim was to establish the *Journal* ‘as the leading international journal of general psychiatry and an essential companion in clinical practice’. To this end he oversaw a process of modernisation, seeking contributions from both internationally recognised and more junior clinicians and academics and improving the rigour of editorial oversight and peer review and the speed of publication. He greatly enjoyed this collaborative creative process but found it difficult to reject good publishable articles for reasons of space.

Greg also undertook research in the UK and with international colleagues, for example the Liverpool-based SQLS project and the international ODIN study. He published 17 books and over 80 articles in books and peer-reviewed journals on general psychiatry, epidemiology and mental health practices in primary care settings. He valued the intellectual discipline and expertise of colleagues from many different branches of medicine, general practice, dentistry, nursing, statistics and publishing. With George Stein he edited *Seminars in General Psychiatry*, published by Gaskell in 1998 and revised in 2007.

He was born in Dundee in 1951, the son of David, a mechanical engineer, and Joan, a department store worker. Greg lived in rural India with his parents from infancy until aged 6, when he returned to Scotland to live with his grandparents. Thereafter he spent his childhood between Dundee and India, seeing his parents only intermittently. At 16 he lived alone in the family house in Forfar. After the family returned from India, his father worked in the Ivory Coast, which Greg visited during vacations. As a teenager and student, he worked as a museum attendant, which fed his lifelong love of the visual arts, as a barman and in a milk bottling plant. These experiences gave Greg a love of travel, an appreciation of style and an independent spirit, all of which were key to his character.

Greg was educated at Lawside Academy in Dundee and subsequently at Edinburgh University, where he initially read psychology but switched courses, graduating in medicine in 1975 and winning his class medal in psychiatry. After graduation Greg worked at Edinburgh Royal Infirmary and Leith Hospital in general medicine. He started specialist training in psychiatry in 1978 on his appointment as Registrar in Psychiatry at the Bethlem and Maudsley Hospitals in London. Apart from 6 months in early 1983 at the Medical Research Council (MRC) Psychiatry Unit in Edinburgh, Greg spent the next 11 years at the Maudsley and King's College Hospitals. He moved into academic psychiatry and became successively joint senior registrar, honorary lecturer, senior lecturer and consultant in the General Practice Research Unit at the Institute of Psychiatry, London, under Michael Shepherd, whom he greatly admired, as he did Edward Hare. Greg eventually became Acting Director of the Unit after Professor Shepherd's retirement in 1988.

In 1989 he was appointed Reader in Psychological Medicine at the University of Wales College of Medicine and Director of the newly opened Academic Department of Psychological Medicine at the North Wales Hospital, Denbigh. He was Professor of Psychiatry at the Royal London Hospital from 1992 to 1994 and Professor of Liaison Psychiatry at the University of Liverpool from 1994 to 2005. He was awarded the Jean Hunter Bequest of the Royal College of Physicians of Edinburgh in 1985 and the Bruce Burns Annual Mental Health Promotion Award in 1990.

On retirement from the NHS in 2005, Greg expanded his successful medico-legal practice as an expert witness in personal injury cases, and remained a part-time consultant in liaison psychiatry at a multidisciplinary clinic for sufferers from atypical facial pain at the Liverpool University Dental Hospital, work he enjoyed immensely and undertook for over 25 years. He also wrote quirky articles on cases of mental illness in history, undertaking research inter alia at Gladstone's Library in Hawarden and the library of his club, the Athenaeum, in London, and via extensive international correspondence with historians and medical academics. He was sceptical about many professional issues, including the merits of ‘care in the community’, the post-Shipman professional supervision system and the influence of administrators rather than the needs of patients in dictating the availability and quality of services.

Greg had remarkable powers of concentration and attention to detail, reflected in his love of the editor's role and his passion for the works of James Joyce. He had numerous outside interests and one of his favourite sayings was that the purpose of work is to go on holiday. He ran, cycled, skied, played tennis and hill-walked in North Wales, Scotland and the French Alps; he baked sourdough bread, planted vegetables and orchards, and made jams, apple juice, cider and wine. He was married firstly to Helen Mackinnon (m. 1972, div. 1980) and from 1984 until his death to Christine (née Lewis), a solicitor to whom he was introduced by their mutual friend Professor Anthony Holland. He is survived by Chris and their four children – Adam, Vicky, Dominic and Alex – and by his grandchild Hector.

